# Single-nucleus transcriptional profiling uncovers the reprogrammed metabolism of astrocytes in Alzheimer’s disease

**DOI:** 10.3389/fnmol.2023.1136398

**Published:** 2023-02-22

**Authors:** Li-Yuan Fan, Jing Yang, Ming-Li Li, Ruo-Yu Liu, Ying Kong, Su-Ying Duan, Guang-Yu Guo, Jing-Hua Yang, Yu-Ming Xu

**Affiliations:** ^1^Department of Neurology, First Affiliated Hospital of Zhengzhou University, Zhengzhou University, Zhengzhou, China; ^2^Clinical Systems Biology Laboratories, Zhengzhou University, Zhengzhou, China; ^3^Academy of Medical Sciences, Zhengzhou University, Zhengzhou, China

**Keywords:** Alzheimer’s disease, single-nucleus transcriptome, astrocyte, metabolism, glutamine, glutamate, neurodegenerative disease

## Abstract

Astrocytes play an important role in the pathogenesis of Alzheimer’s disease (AD). It is widely involved in energy metabolism in the brain by providing nutritional and metabolic support to neurons; however, the alteration in the metabolism of astrocytes in AD remains unknown. Through integrative analysis of single-nucleus sequencing datasets, we revealed metabolic changes in various cell types in the prefrontal cortex of patients with AD. We found the depletion of some important metabolites (acetyl-coenzyme A, aspartate, pyruvate, 2-oxoglutarate, glutamine, and others), as well as the inhibition of some metabolic fluxes (glycolysis and tricarbocylic acid cycle, glutamate metabolism) in astrocytes of AD. The abnormality of glutamate metabolism in astrocytes is unique and important. Downregulation of *GLUL* (*GS*) and *GLUD1* (*GDH*) may be the cause of glutamate alterations in astrocytes in AD. These results provide a basis for understanding the characteristic changes in astrocytes in AD and provide ideas for the study of AD pathogenesis.

## Introduction

Alzheimer’s disease (AD) is the most common neurodegenerative disease in the world. The clinical features include memory loss, and the pathological features include amyloid-β (Aβ) accumulation, neurofibrillary tangle formation (accumulation of p-tau), extensive neuroinflammation, and synaptic toxicity. Unfortunately, there is currently no cure for AD, and the development of drugs, such as those targeting Aβ has not been successful, possibly because the focus of the field is narrow. Therefore, new concepts of AD are needed.

It is becoming increasingly clear that, in addition to neurons, glial cells in the brain respond to Aβ and tau pathology (De Strooper and Karran, 2016). Genetic studies have shown that the risk of late-onset AD is associated with genes that are primarily expressed by glial cells, such as *APOE*, *APOJ*, and *SORL* (Wang et al., 2016; Almeida et al., 2018; Shi and Holtzman, 2018). This view has shifted the focus of research from neurons to glial cells and neuroinflammation. Astrocytes maintain homeostasis in the brain by providing nutritional and metabolic support to neurons. They circulate neurotransmitters, stimulate synaptic transmission, and form part of the blood–brain barrier (Verkhratsky and Nedergaard, 2018). Astrocytes atrophy occurs in many central nervous system (CNS) disorders (e.g., AD, frontotemporal dementia, epilepsy, and schizophrenia; Pekny et al., 2016). Molecular studies have shown that astrocytes regulate vascular units and influence the clearance of tau and Aβ (Rasmussen et al., 2018). The importance of astrocyte function and energy homeostasis cannot be ignored in the normal functioning of neurons and the brain.

Single-nucleus transcriptomics allows tracking of the fate of individual astrocytes and describes the state of healthy and pathological cells at different stages of AD. Our present study showed the metabolism of different cells in the prefrontal cortex (PFC) of patients with AD by integrating a single-nucleus sequencing (snRNA-seq) database. Our analysis results illustrated the unique metabolic state of astrocytes in AD brains. Some of important metabolites, such as 2-oxoglutarate (2OG), acetyl-coenzyme A (CoA), aspartate, pyruvate, and glucose-6-phosphate (G6P), are consumed or accumulated. Glutamate metabolism is an important and unique metabolic process in astrocytes. In the astrocytes in AD brains, glutamate accumulates, while levels of the glutamine and 2OG are downregulated. This reprogrammed metabolism in astrocytes may result from the downregulation of *GLUL* and *GLUD1*. These findings suggested abnormalities in glutamate and energy metabolism in astrocytes of AD brains, providing metabolic evidence for further research on the pathogenesis of AD.

## Materials and methods

### Resources of snRNA-seq data and pre-processing analysis

The snRNA-seq datasets (raw or gene expression matrices) of the human PFC used in this study were downloaded from the NCBI Gene Expression Omnibus database (GEO)^1^ under accession numbers GSE157827 (Lau et al., 2020), GSE174367 (Morabito et al., 2021), containing 24 AD and 17 control samples in total. After merging all the datasets, cells with less than 200 unique molecular identifiers (UMIs), more than 5,000 UMIs, or mitochondrial counts greater than 20% were filtered out. Genes expressed in fewer than three cells were also filtered out. Seurat (version 4.0)^2^ was used for a wide variety of single-cell analyses, including normalization, scaling, batch correction, dimensionality reduction, clustering, and visualization. We used the harmony package^3^ for the batch correction. The expression of known marker genes in the CNS was used as a reference for the annotation of different cell types. All statistical analyses were conducted using the R software (version 4.0.2).

### Cell type-specific metabolic analysis

Single-cell flux estimation analysis (scFEA; Alghamdi et al., 2021) was used to identify the single-cell metabolic flux profiles. A total of 168 metabolic modules were directly downloaded from the algorithm’s official GitHub page.^4^ Using default parameters, the FindMarkers function (the nonparametric Wilcoxon rank sum test) was used for differential expression analysis. Differential expression genes with p_val_adj < 0.05 were then selected for futher analysis. Using default parameters, the Monocle2 (version 2.22.0; Trapnell et al., 2014) algorithm was used to construct a single-cell pseudotime trajectory. We used the 2,000 most highly variable genes for analysis and identified 5 distinct cell states. State-specific genes were identified using differentialGeneTest, which compares the expression of genes between each state and the remaining four states. The trajectories were constructed using DDRTree.

### Scwgcna and gene set enrichment analysis

The WGCNA R package (Langfelder and Horvath, 2008) was used to perform a weighted correlation network analysis (*WGCNA*) and module preservations. The 5,000 most highly variable genes and the differentially expressed enzymes were used. A softPower of 6 was selected as the soft-threshold parameters to ensure a signed co-expression gene network. After filtering, there left a total of 5,007 genes. The correlation coefficient calculation process uses the Spearman correlation coefficient method of the Cor function. To identify the genes in the yellow module that were regulated in AD, gene set enrichment analysis (GSEA) and hypergeometric tests with the clusterProfiler R package were used for functional enrichment analysis.

### Gene regulatory network analysis

We used the pyscenic (Aibar et al., 2017) based on hg19-tss-centered-10 kb-10species databases to analyze the enrichment of transcriptome factors in astrocytes. For the single-cell regulatory network inference and clustering (SCENIC) workflow, default parameters were used, and the raw count matrix from all samples was used as the input. Using GENIE3, we calculated co-expression modules and evaluated the correlation between transcription factors (TFs) and their target genes. RcisTarget reveals TFs with direct targets (regulons).

## Results

### Integrated dataset revealed the single-nucleus transcriptional states of Alzheimer’s disease

**Figure 1:**
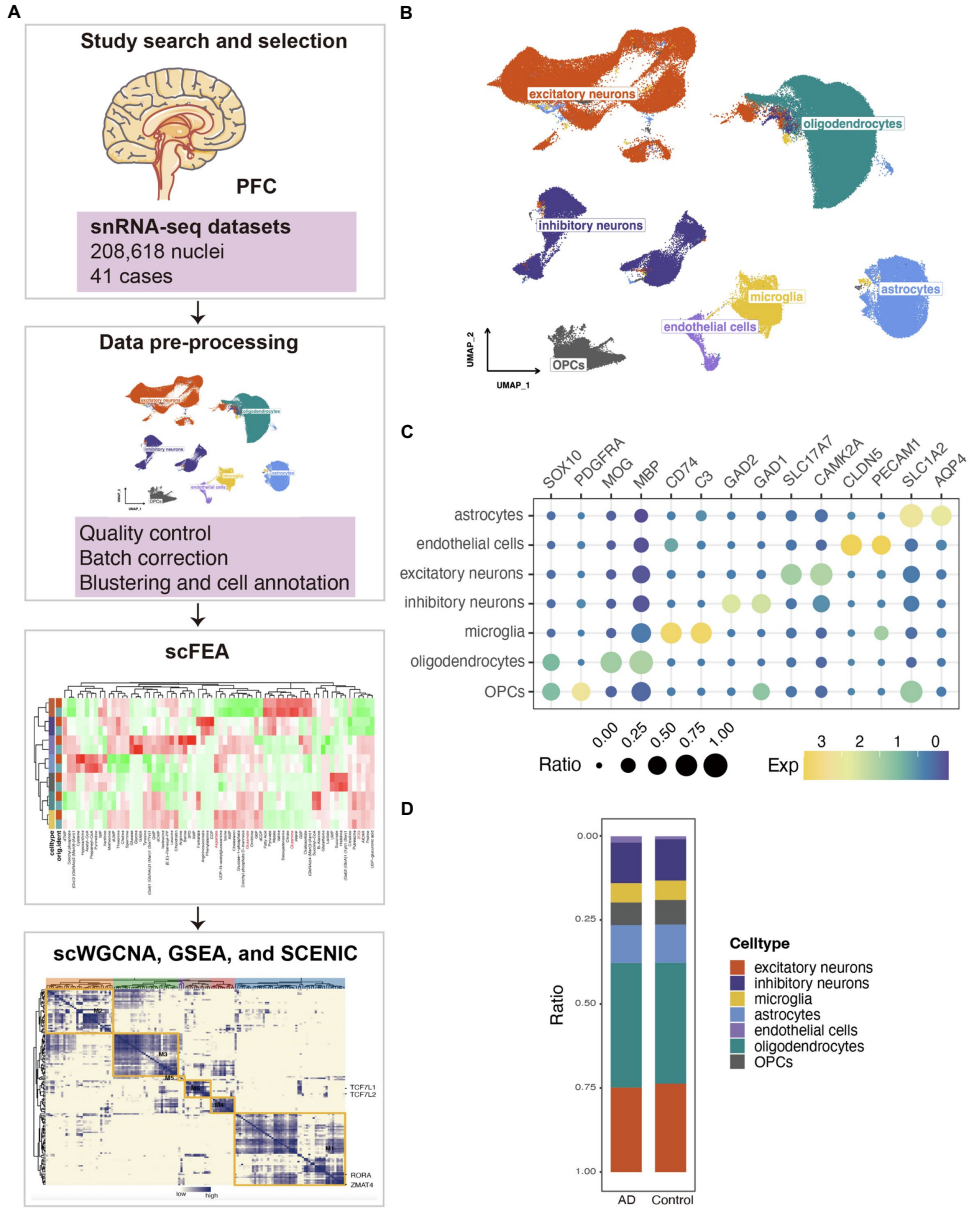
Single-nucleus transcriptional landscape of AD. **(A)** Flow chart of the experimental analysis. **(B)** Uniform manifold approximation and projection (UMAP) representation of the landscape of different cell types. **(C)** Features plots for the merged single-nucleus sequencing (snRNA-seq) data demonstrated the expressions of the markers in the different nuclei clusters. **(D)** Stacked bar plots of the differing cell-type proportions in emerged datasets.

We integrated snRNA-seq datasets of the human PFC for in-depth analysis (Figure 1A). The neuropathological characterization and clinical history of the patients and controls was in the attachment (Supplementary Table S1). After quality control, batch correction, unsupervised nucleus clustering, differential expression analysis, and classification, 208,618 nuclei were retained in the merged snRNA-seq data. In the uniform manifold approximation and projection (UMAP) space, we profiled nine major cell types using Seurat’s data integration pipeline (Stuart et al., 2019; Figure 1B). We profiled the major cell types in the PFC: astrocytes (*AQP4*^+^, *SLCA2*^+^), endothelial cells (*PECAM1*^+^, *CLDN5*^+^), excitatory neurons (*CAMK2A*^+^, *SLC17A7*^+^), inhibitory neurons (*GAD2*^+^, *GAD1*^+^), microglia (*C3*^+^, *CD74*^+^), oligodendrocytes (*MBP*^+^, *MOG*^+^), and oligodendrocyte precursors (OPCs; *PDGFRA*^+^, *SOX10*^+^; Figure 1C). We estimated cell-type proportions and observed that the proportions of various cells in the AD group did not change significantly compared with the control group (Figure 1D).

### Changes in metabolic signatures of the prefrontal cortex of Alzheimer’s disease

**Figure 2:**
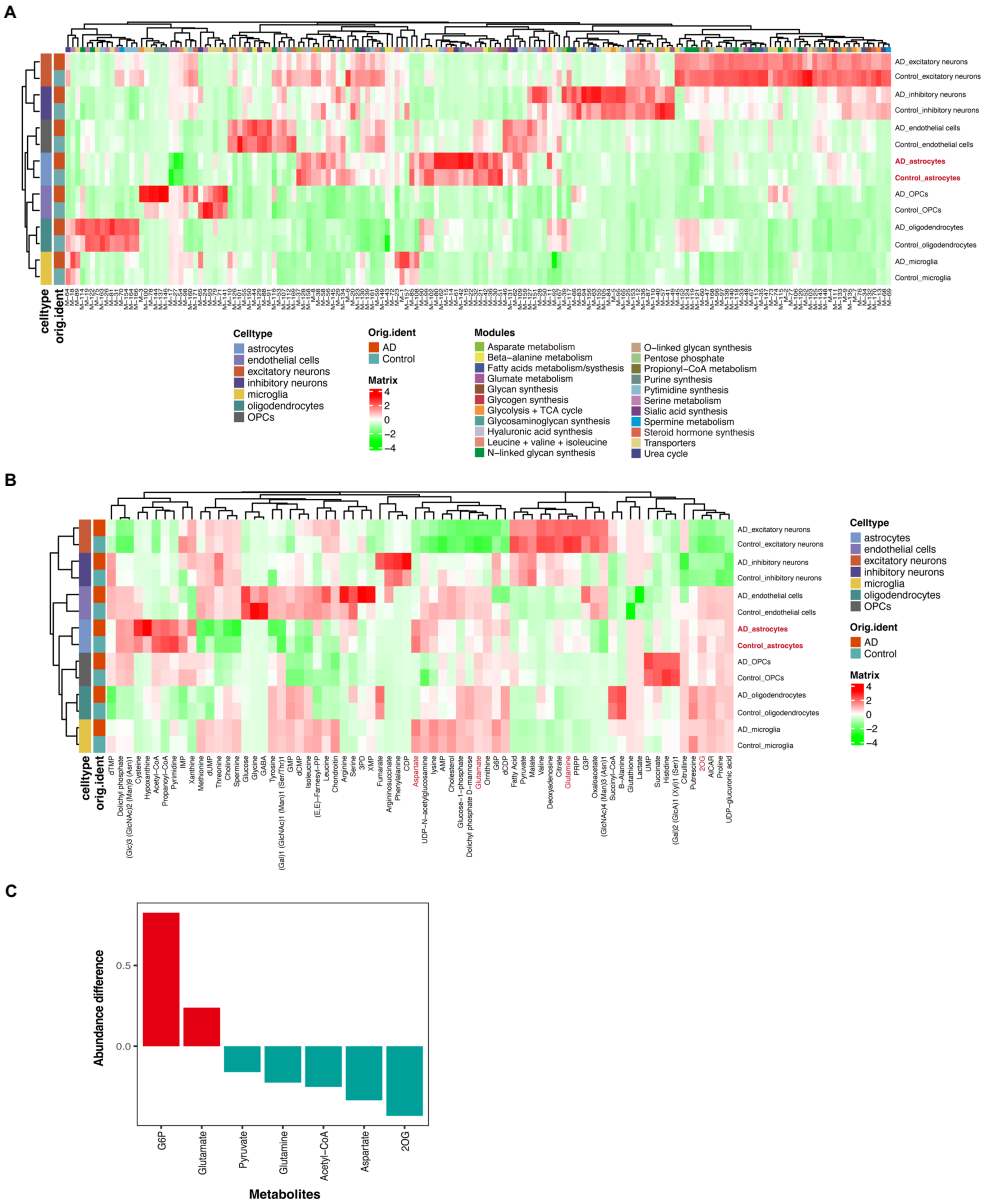
Single-cell metabolic flux mapping of prefrontal cortex (PFC) revealed metabolism heterogeneity of AD. **(A)** Profile of the predicted flux of metabolic modules. **(B)** Profile of the predicted metabolites. **(C)** The statistical chart of some important metabolites. The ordinate represents the ratio of the metabolite contents of the disease group to the control group.

To identify the unique metabolism in AD, we used a new computational method, scFEA (Alghamdi et al., 2021), to infer the cell flux set from the snRNA-seq data. We noticed that different cell types had different main metabolic fluxes (Figure 2A; Supplementary Table S2). We observed that most metabolic flux in the astrocytes of patients with AD was dysregulated, especially the glycolysis + tricarbocylic acid (TCA) cycle, glutamate metabolism, and transporters (Figure 2A). Most of the metabolites in the astrocytes of patients with AD were depleted, such as acetyl-CoA, aspartate, pyruvate, 2OG (also known as α-ketoglutaric acid [α-KG]), and glutamine, compare with the control group (Figures 2B,C). G6P and glutamate accumulated in the AD group while compared with that in the control group (Figures 2B,C). We also detected the key genes that played an important role in each metabolic module (Supplementary Table S3).

### Reprogrammed glutamate metabolism of astrocytes of Alzheimer’s disease

**Figure 3:**
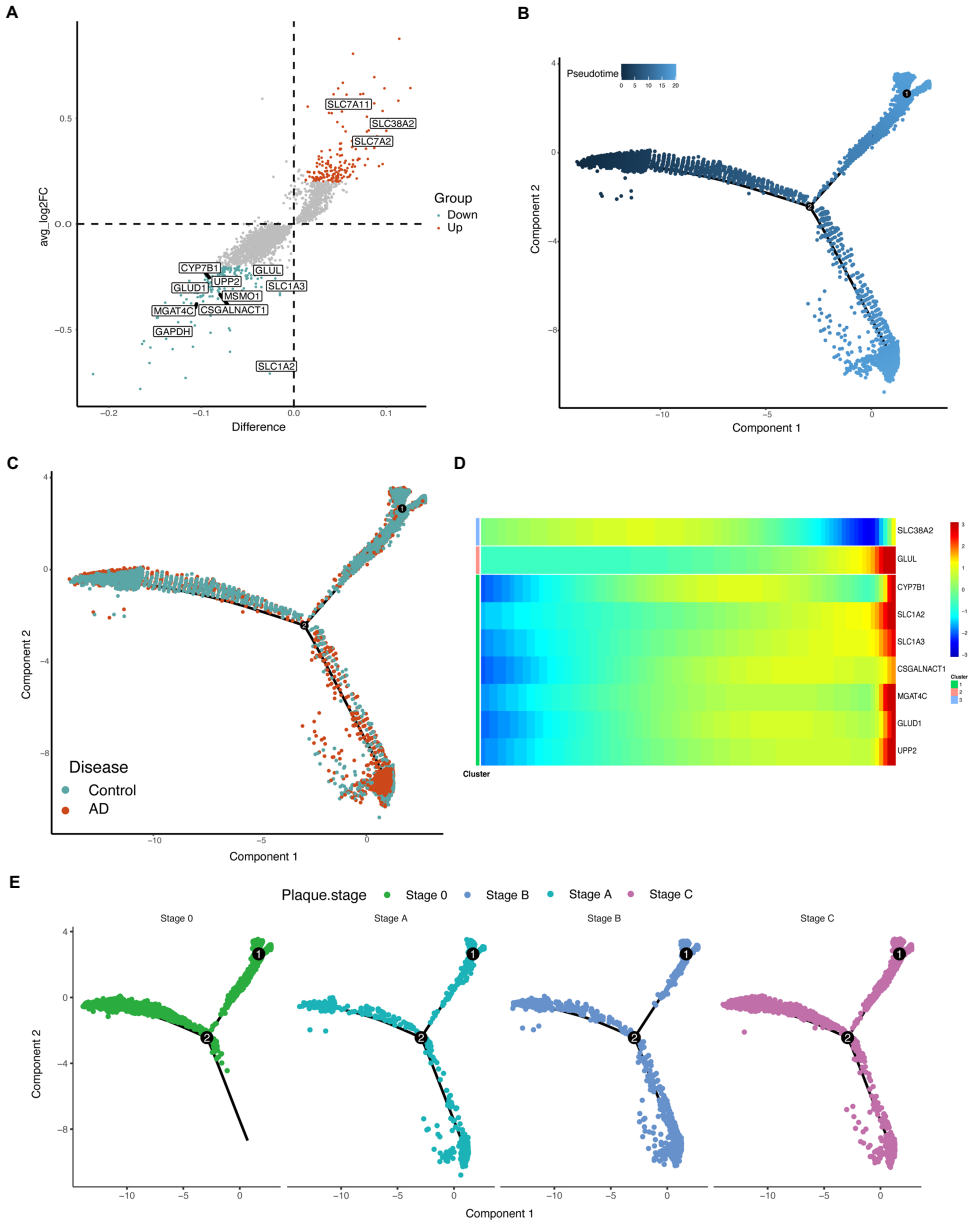
Demonstration of differentially expressed genes in astrocytes. **(A)** Volcano map revealing differences in genes expression in astrocytes. The nonparametric Wilcoxon rank sum test wae used for differential expression analysis. Differential expression genes with p_val_adj < 0.05 were then selected for analysis. The metabolism-associated enzymes and transporters that differ significantly are highlighted in the figure (take the intersection of differentially expressed genes and metabolism-related genes). **(B)** Cell trajectories are calculated based on pseudotime values. Pseudotime 0–20 represents the stages of progression in different states of the cell. Component represents the dimensions on the Monocle nonlinear dimensionality reduction space. **(C)** Cell trajectories are calculated based on pseudotime values, split by disease. Component represents the dimensions on the Monocle nonlinear dimensionality reduction space. **(D)** Heatmap of some enzymes and transporters expressed differently over pseudotime. **(E)** Cell trajectories are calculated based on pseudotime values, split by plaque stages of patients with AD and controls. Component represents the dimensions on the Monocle nonlinear dimensionality reduction space.

Differentially expressed genes in astrocytes were identified, and some differentially expressed metabolism-related enzymes were observed (Figure 3A; Supplementary Table S4). Glutamate metabolism in astrocytes is an important and characteristic metabolic process. scFEA predicted two reduced glutamate metabolism fluxes, M48 (glutamate→glutamine) and M51 (glutamate→2OG), in astrocytes of the AD group compared with that in the control group (Figure 2A). The metabolites of M48 (glutamine) and M51 (2OG) metabolites were also reduced in the AD group (Figure 2B). Interestingly, both the enzymes *GLUL* and *GLUD1*, which regulate M48 and M51, respectively, were downregulated in the AD group (Figure 3A; Supplementary Table S4). These results suggested that alterations in glutamate metabolism in astrocytes of AD may be due to the abnormal expressions of *GLUL* and *GLUD1*. Additionally, M85 (aspartate_in→aspartate) flux, the metabolites (aspartate), and flux-related transporter (*SLC1A3*) were also significantly downregulated (Figures 2, 3A; Supplementary Tables S3, S4).

To effectively identify possible astrocytes fates and time-regulated genes, Monocle2 (Trapnell et al., 2014) was used to perform pseudotemporal ordering of cells to understand the overall “trajectory” of gene expression changes and place each cell in the appropriate position in the trajectory. The trajectories revealed that the astrocytes of in the AD group and control group were clearly on different branches during pseudotime (Figures 3B,C), which means the different expression patterns of genes in the two groups. The number of cells in the AD group gradually increased over pseudotime (Figures 3B,C). Some regulated enzymes and transporters, *GLUL*, *CYP7B1*, *SLC1A2*, *SLC1A3*, *CSGALNACT*, *MGAT4C, GLUD1*, and *UPP2* were upregulated over pseudotime, while the trend of *SLC38A2* was the opposite (Figure 3D; Supplementary Table S5). In the different plaque stages of patients with AD, the distribution of astrocytes during the pseudotime was also different (Figure 3E).

**Figure 4:**
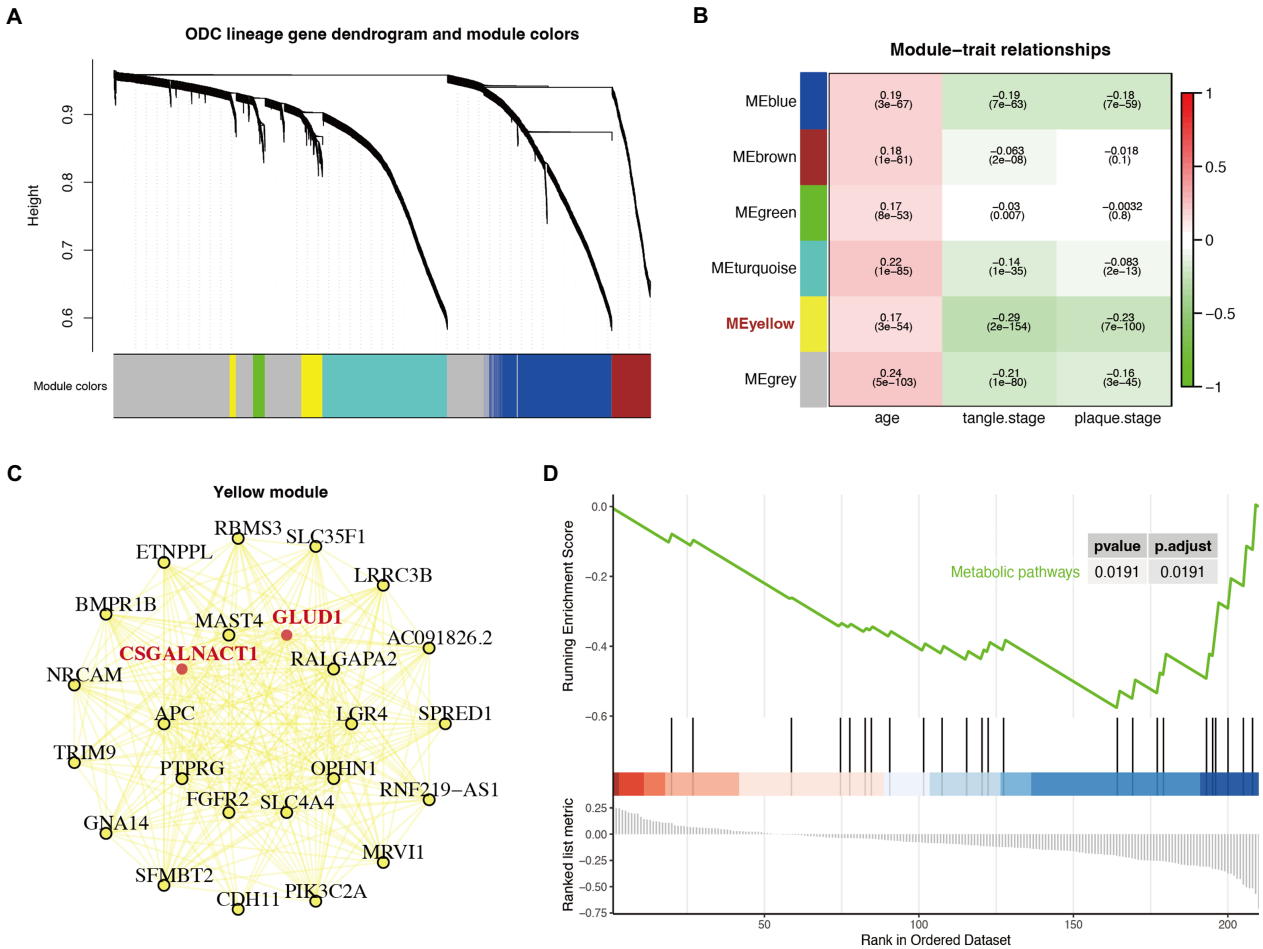
Weighted correlation network analysis (WGCNA) and gene set enrichment analysis (GSEA) analysis identified a particular module associated with metabolic shift**. (A)** Gene dendrogram with clustering based on consensus topological overlap. Lower color row: consensus modules following merging of similar modules. Five consensus non-gray modules across all samples were identified (represented by different colors). **(B)** Heatmap of the correlation of the modules with age, plaque stage, and tangle stage. This calculation process uses the Spearman correlation coefficient method of the Cor function. The correlation coefficient and *p*-value are shown. **(C)** The module-network analysis of the yellow module. Bold and marked in red are differentially expressed metabolism-related genes. **(D)** GSEA diagram of the genes in the yellow module. The results showed that the genes in the yellow module were closely related to metabolism pathway (*p* < 0.05).

WGCNA is to explore whether there is a pattern of co-expression of each gene between samples. A certain group of co-expressed genes were divided into a module according to a certain value. To further determine changes in metabolism, we selected the first 5,000 highly variable genes of astrocytes for WGCNA analysis (Langfelder and Horvath, 2008) and identified 5 consensus non-gray modules across all samples (Figure 4A). Among these non-gray modules, the yellow module had the highest absolute correlation values with the plaque and tangle stages (Figure 4B). The yellow module consisted of 253 genes, including most of the differentially expressed enzymes and transporters (*GLUD1*, *GLUL*, *SLC1A2*, *MGAT4C*, *CSGALNACT1*, *SLC1A3*, *CYP7B1*, and *UPP2*; Supplementary Table S6). Further module-network analysis showed that the differentially expressed enzymes *GLUD1* and *CSGALNACT1* were the core of the yellow module (Figure 4C). We further performed GSEA analysis on the yellow module, and the KEGG results indicated that the metabolic pathways were closely related to the genes involved in the module (Figure 4D).

### Transcriptional dynamics and the regulators of astrocytes in Alzheimer’s disease

**Figure 5:**
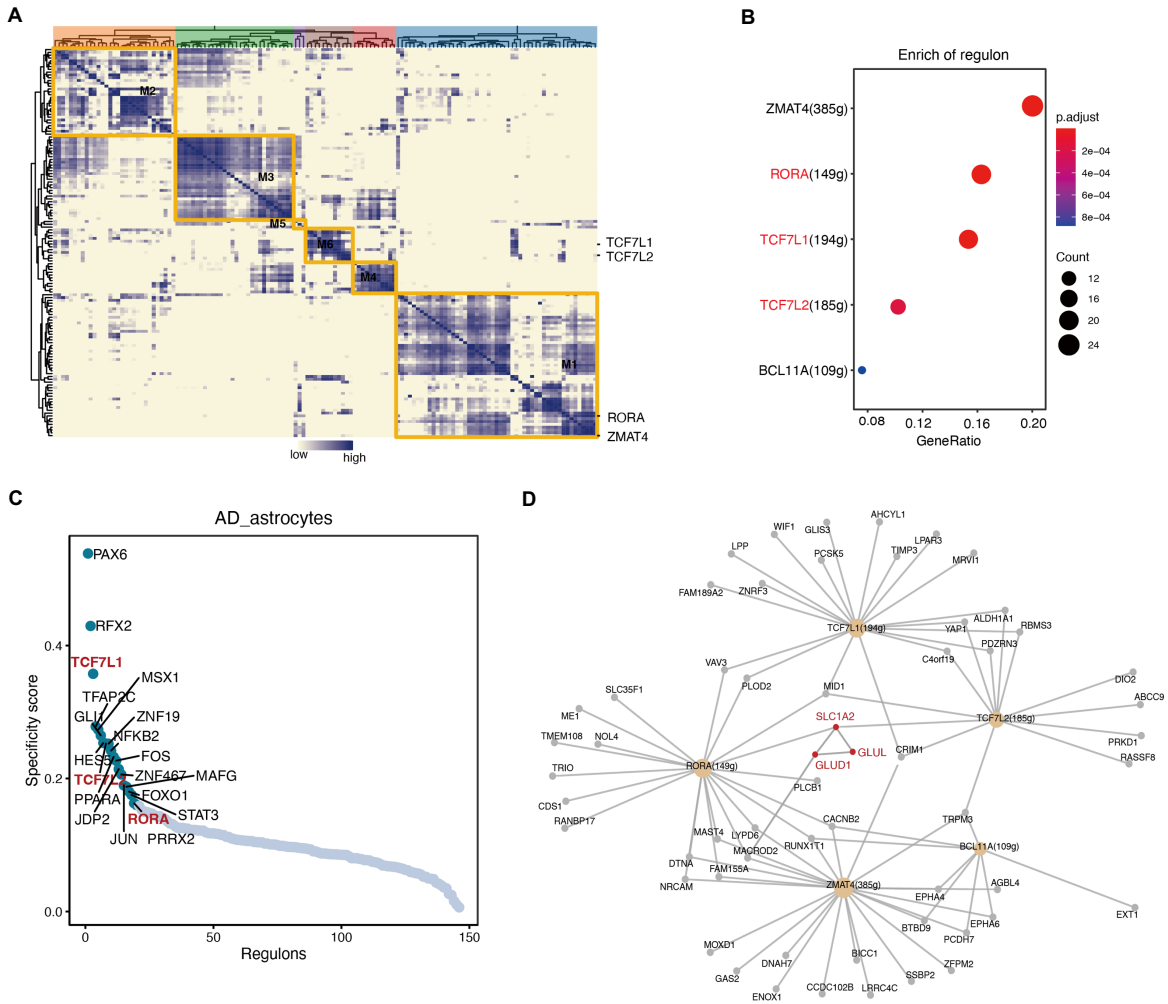
Single-cell regulatory network inference and clustering (SCENIC) analysis identified transcriptional regulatory networks that related to the reprogrammed metabolism in astrocytes. **(A)** Hierarchical clustering heatmap of the discovered regulons, and 5 main modules are represented. **(B)** Transcriptional regulators that are significantly enriched in the yellow module. **(C)** The specificity scores of regulons in astrocytes. The top 20 genes with higher activity are noted. **(D)** The network of the main regulons in the yellow module, and the interaction diagram between some important enzymes (red font).

We then examined whether the alterations in any transcription factors (TFs) were included in the astrocytes by performing SCENIC. We performed a hierarchical clustering analysis of the transcriptional regulators and obtained five modules (Figure 5A; Supplementary Table S7). Using the enrichment analysis algorithm and hypergeometry tests, we identified some transcriptional regulators that had the greatest impact on the yellow module (Figure 5B). Among them, *TCF7L1*, *TCF7L2*, and *RORA* showed high specificity for astrocytes (Figure 5C). Interestingly, after visualizing the TFs and their targets, we found that both *RORA* and *TCF7L2* regulated the transcription of the differentially expressed gene *SLC1A2* (Figure 5D), which was downregulated in astrocytes of AD (Figure 3A). The interaction network showed that *SLC1A2* interacts with the differentially expressed genes *GLUL* and *GLUD1* (Figure 5D).

## Discussion

Recent proteomics studies on human AD brain samples have found a strong correlation between the severity of AD pathology and the proteins associated with astrocytes metabolism (Johnson et al., 2020). By integrating multiple single-cell databases, we presented the metabolic profiles of various cell types in the PFC of patients with AD. Astrocytes of interest exhibited significantly abnormal metabolism, especially the cell-specific glutamate metabolism. Astrocytes are necessary for maintaining homeostasis in the brain and protecting neurons. Under different pathological conditions, including AD, they react, leading to neuroinflammation and neurodegeneration. A direct link has been reported between astrocytes and amyloid plaques (Frost and Li, 2017; Bennett and Viaene, 2021). Glutamate is the primary excitatory neurotransmitter in the brain. Glutamate circulates extensively between neurons and astrocytes, a process known as the glutamate-glutamine cycle. Glutamate recirculation is closely related to brain energy metabolism and is necessary for the maintenance of glutamatergic neurotransmission. Disruption of glutamate clearance leads to neuronal hyperstimulation and excitotoxicity (Andersen et al., 2021b).

Approximately 80% of all glutamate taken up by astrocytes is converted to glutamine (Hertz and Rothman, 2016). This process is catalyzed by the enzyme glutamine synthetase (*GLUL*), which is specifically expressed in astrocytes (Norenberg and Martinez-Hernandez, 1979). In our study, the transcription level of *GLUL* in astrocytes of the brains of patients with AD was downregulated, and the conversion of glutamate to glutamine was inhibited. At the same time, glutamate accumulated in astrocytes, which we speculate may be responsible for the overstimulation and excitation of neurons in the brains of patients with AD.

Glutamate is associated with cellular energy metabolism *via* the TCA cyclic intermediate 2OG, which is catalyzed by glutamate dehydrogenase 1 (*GLUD1*; Sonnewald, 2014). Multiple studies have shown that the hippocampal expression of GLUD1 is a strong determinant of memory decline in aging and AD (Ciavardelli et al., 2010; Neuner et al., 2017). GLUD1-deficient astrocytes produce less ATP during glutamate uptake (Hohnholt et al., 2018). Studies have reported that when glutamate accumulates, astrocytes reduced glutamine synthesis and increase the oxidative metabolic capacity of glutamate in the TCA cycle (McKenna et al., 1996). However, in our study, although the transcription of *GLUD1* was inhibited, the conversion between glutamate and 2OG was downregulated. These results supported the importance of *GLUD1*-mediated glutamate metabolism for energy metabolism and proved that the pathways of glutamate metabolism in the astrocytes of AD brains are abnormal, resulting in glutamate accumulation.

A study found a decrease in selectivity for astrocytes aspartate synthesis in 5xFAD mouse hippocampal slices (Andersen et al., 2021a), which was consistent with our findings on human PFC. Intriguingly, neuron-derived aspartic acid is a prominent nitrogen donor for glutamate and glutamine synthesis in astrocytes (Pardo et al., 2011), and the neuronal uptake of glutamate is also crucial for maintaining neuronal aspartic acid pools (McNair et al., 2019). Our study also strongly suggests abnormalities in aspartate metabolism and glutamate metabolism, indicating that astrocytes-specific defects in AD disrupted aspartate and glutamate homeostasis. In addition, this study demonstrated that altered astrocytic glutamine synthesis has functional consequences for neurons in AD, directly impairing neuronal GABA synthesis in the brain slices of the AD 5xFAD mouse model (Andersen et al., 2021a). However, our study did not find any significant changes in GABA metabolism in astrocytes or neurons.

The mechanisms underlying neuronal loss are complex and not yet fully being understood. The excitotoxicity of glutamate has been hypothesized to lead to brain pathology (Beal, 1992). The study of glutamate metabolism may provide new metabolic insights into AD. We also acknowledged several potential limitations of this current study. First, our analysis was based on transcriptomics; thus, the expression of relevant proteins and metabolites still needs further validation, which is also our ongoing research. Second, the brain is an extremely complex system in which various nerve cells interact with each other. Therefore, further research on the metabolic communications between astrocytes and other cells is required.

## Data availability statement

The datasets presented in this study can be found in online repositories. The names of the repository/repositories and accession number(s) can be found in the article/Supplementary material.

## Author contributions

L-YF and G-YG collected and analyzed the data. L-YF wrote the manuscript. JY, M-LL, R-YL, YK, and S-YD conceptualized and oversaw the study and data analysis. J-HY and Y-MX provided funding supports. All authors have read and approved the final manuscript.

## Funding

This work was supported by the National Natural Science Foundation of China (Grant U1904207); National Key R&D Program of China (Grant 2017YFA0105003); Non-profit Central Research Institute Fund of Chinese Academy of Medical Sciences (Grant 2020-PT310-01); Innovative and Scientific and Technological Talents Training Project of Henan Province (Grant YXKC2021062).

## Conflict of interest

The authors declare that the research was conducted in the absence of any commercial or financial relationships that could be construed as a potential conflict of interest.

## Publisher’s note

All claims expressed in this article are solely those of the authors and do not necessarily represent those of their affiliated organizations, or those of the publisher, the editors and the reviewers. Any product that may be evaluated in this article, or claim that may be made by its manufacturer, is not guaranteed or endorsed by the publisher.

## Supplementary material

The Supplementary material for this article can be found online at: https://www.frontiersin.org/articles/10.3389/fnmol.2023.1136398/full#supplementary-material

## Footnotes

^1^ https://www.ncbi.nlm.nih.gov/geo/

^2^ https://satijalab.org/seurat/

^3^ https://github.com/immunogenomics/harmony

^4^ https://github.com/changwn/scFEA
